# Modified technique for double J stent removal

**DOI:** 10.1590/0100-3984.2020.0009

**Published:** 2021

**Authors:** Thiago Franchi Nunes, Tiago Kojun Tibana, Rômulo Florêncio Tristão Santos, Riccardo Inchingolo

**Affiliations:** 1 Hospital Universitário Maria Aparecida Pedrossian da Universidade Federal de Mato Grosso do Sul (HUMAP-UFMS), Campo Grande, MS, Brazil.; 2 Interventional Radiology Unit, “F. Miulli” Regional General Hospital, Acquaviva dele Fonti (BA), Italy.; 3 Radiology Department, King’s College Hospital, London, UK.

## INTRODUCTION

Interventional procedures have been used with increasing frequency for the diagnosis and treatment of urological disorders^(1-5)^. The classic method for removing double J (DJ) stents consists of a retrograde cystoscopic procedure involving the use of forceps^(6-9)^. However, retrograde removal may be difficult or not feasible in cases of DJ stent migration or abnormal ureter anatomy due to deviation or previous urinary tract surgery^(10)^. Some techniques to overcome these challenges have been described^(7-11)^. Kim et al.^(12)^ described a DJ stent removal technique using the combination of a snare and a guidewire.

The objective of this article is to describe a modified technique using only a combination of guidewires, replacing the use of a snare with that of a looped guidewire, together with a 9F vascular introducer and a 5F pigtail catheter, which could be equally effective and could reduce costs. Future studies with larger numbers of cases could compare these approaches in terms of their effectiveness and cost-benefit.

## PROCEDURE

### Percutaneous transrenal removal

**Figure 1:**
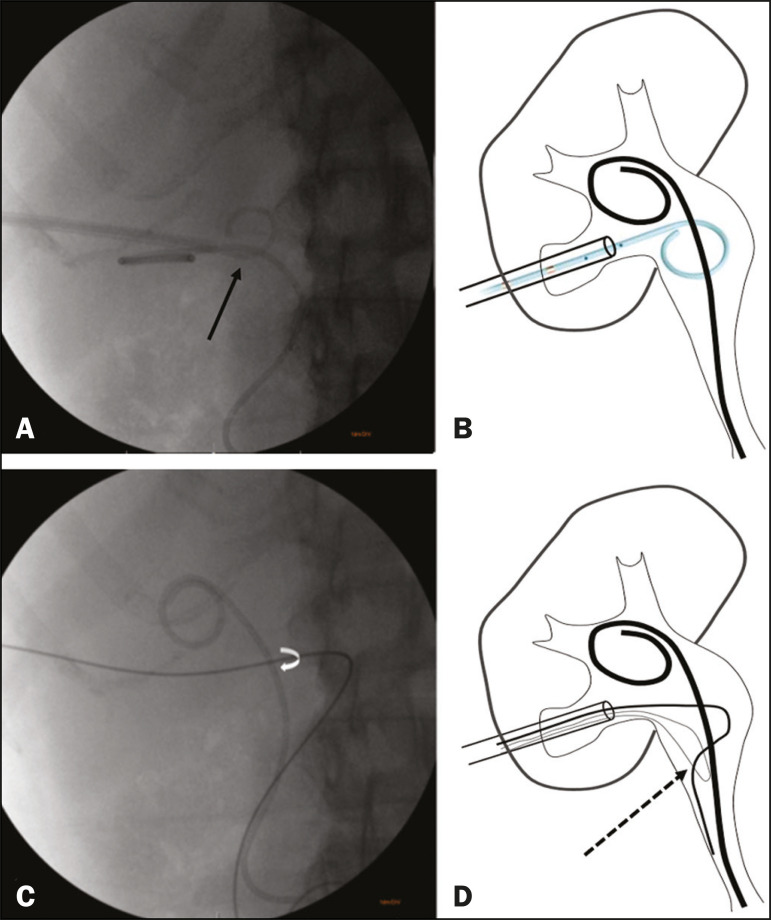
**A,B**: Passage of a 5F pigtail catheter fully encompassing the DJ stent (arrow). **C**: Hydrophilic guidewire, after removal of the pigtail catheter, encompassing the DJ stent (curved arrow). **D**: Doubled 0.014’’ guidewire with the 0.035’’ hydrophilic guidewire inside the loop (arrow).

Percutaneous access to the renal collecting system is typically achieved under ultrasound guidance with the patient in the lateral oblique position. Local anesthesia with 2% lidocaine (10 mL) is administered under conscious sedation. Transhepatic percutaneous puncture is then performed with a Chiba 17G × 10.6 cm needle (Argon Medical Devices, Frisco, TX, USA) and a 9F vascular introducer (Radifocus Introducer II; Terumo, Tokyo, Japan). Antegrade pyelography with injection of iodinated contrast is performed to visualize the anatomy of the collecting system and the position of the previously inserted DJ stent. A 5F × 90 cm pigtail catheter is then inserted into the renal pelvis. Through rotational maneuvers, the pigtail catheter is positioned to fully encompass the DJ stent (Figures 1A and 1B). Subsequently, a 0.035’’ × 180 cm hydrophilic guidewire is passed through the pigtail, after which it is retracted in such a way that the circumferential wrapping of the DJ stent by the hydrophilic guidewire is maintained. A 0.014’’ × 300 cm guidewire is doubled and inserted in the form of a lasso. The hydrophilic guidewire is positioned inside the “lasso” formed by the 0.014’’ guidewire (Figures 1C and 1D). The 9F introducer is advanced to the captured segment of the DJ stent, which is then removed by the introducer together with the two guidewires. After the partial removal of the DJ stent through the external orifice of the introducer, the hydrophilic guidewire is again inserted up to its distal part. Finally, a new DJ stent is inserted by a technique previously described^(1)^.

### Transurethral removal

Transurethral access initially requires good asepsis of the vulvar region in women and of the penile region in men. Carefully and under fluoroscopy, a 9F valved introducer is passed into the bladder and then distended with approximately 200 mL of saline solution and 5% iodinated contrast, to ensure the safety of the procedure. Bladder anatomy and the position of the previously inserted DJ stent are evaluated under fluoroscopy. The same transrenal capture technique described above is performed. After the old DJ stent has been removed and the guidewire has been inserted into the renal pelvis, the new DJ stent is inserted. A bladder probe is left in place for the first 24 h to assess urine output and the functioning of the DJ stent.
